# Preliminarily evaluation the safety and immunogenicity of tetanus, reduced diphtheria and acellular pertussis (five components) combined vaccine, adsorbed (Tdcp) in participants aged 6 years and above: a blinded and randomised, and controlled phase I clinical trial

**DOI:** 10.3389/fimmu.2025.1616574

**Published:** 2025-09-05

**Authors:** Xiaoyu Liu, Chen Wei, Haitao Huang, Jingxuan Wan, Yajun Li, Feiyu Wang, Siwen Li, Ying Wang, Xuewen Wang, Xue Wang, Xiuwen Sui, Jinbo Gou, Tao Zhu, Xiao Ma, Weijun Hu

**Affiliations:** ^1^ Department of Immunization Program, Shaanxi Provincial Center for Disease Control and Prevention, Xi’an, China; ^2^ National Institutes for Food and Drug Control, Beijing, China; ^3^ CanSino Biologics Inc., Tianjin, China; ^4^ Shanghai Imstat Medical Technology Co., Ltd, Shanghai, China

**Keywords:** pertussis, Tdcp vaccine, safety, immunogenicity, phase I, clinical trial

## Abstract

Pertussis remains a significant global public health challenge, particularly in China, where no licensed pertussis-containing vaccines are available for individuals aged 6 years and older. This blind, randomised, and controlled phase I clinical trial evaluated the safety and immunogenicity of the Tetanus, Reduced Diphtheria, and Acellular Pertussis (Five Components) Combined Vaccine, Adsorbed (Tdcp). Participants aged 6 years and above were divided into three age subgroups (6–11, 12–17, and ≥18 years) and randomised to receive Tdcp or control vaccines (PPV23 or DT). Safety endpoints included the incidence of adverse reactions within 30 days post-vaccination, while immunogenicity was assessed through seroconversion rates and geometric mean concentrations (GMCs) of anti-DT, TT, PT, FHA, PRN, and FIM 2&3 antibodies at 30 days post-vaccination. Among 178 participants, the incidence of total adverse reactions in the Tdcp group was 54.62%, primarily local reactions such as pain and itching, with no significant differences compared to controls (p > 0.05). Immunogenicity analysis revealed robust immune responses in the Tdcp groups, with seroconversion rates for pertussis-related antigens exceeding 75% in all age groups. The GMCs for anti-PT antibodies reached 125.60 IU/mL, 150.28 IU/mL, and 131.14 IU/mL in the ≥18 years, 12–17 years, and 6–11 years of Tdcp groups, respectively. Overall, the Tdcp vaccine demonstrated a good safety profile and robust immunogenicity in participants aged 6 years and above. The inclusion of five pertussis antigens, particularly PT, elicited strong immune responses, supporting its potential as an effective booster vaccine for adolescents and adults.

**Clinical trial registration:**

https://clinicaltrials.gov/, identifier NCT06056050.

## Introduction

Pertussis, an acute and highly contagious respiratory disease caused by Bordetella pertussis, remains a significant global public health challenge despite reduced incidence through vaccination (1). Initial symptoms such as runny nose, low-grade fever, and mild cough can progress to severe coughing fits, vomiting, and respiratory distress. Infants, who may not exhibit typical coughing, face life-threatening complications like apnea (2). Over the past few years, during and after the COVID-19 pandemic, reported pertussis cases were lower than usual. This decline was likely due to pandemic measures like masking and remote learning, which reduced transmission. However, the U.S. is now returning to pre-pandemic patterns, with more than 10,000 cases typically reported each year. In 2024, pertussis cases increased across the U.S., showing a return to normal trends. Preliminary data as of December, 2024, revealed that cases were more than six times higher compared to the same period in 2023. The number of cases in 2024 also exceeded those reported in 2019, before the pandemic (3). According to data from the China CDC in 2024, there were 494,321 reported cases of pertussis, which is twice the total number of cases from 2000 to 2023 (215,699 cases). The number of deaths from the disease reached 31 cases. The high-incidence group was school-age children. China has witnessed a major outbreak of pertussis (4). One study investigated 14,874 PCR-confirmed cases of pertussis in children at the sentinel hospitals in five Chinese provinces from 1 January to 30 September 2024, and children aged 6–10 accounted for 52.32% of cases (5). Moreover, in China, systematic surveillance by the Tianjin Center for Disease Control and Prevention revealed four transmission clusters (household, school, hospital, village), with household transmission dominating (85.33%). Between 2010 and 2012, household clusters showed an average infection rate of 77.88%, with 24 families experiencing 100% attack rates. Adult-to-infant transmission (67.19%) was the primary pattern, with parents identified as the main source of infant infections (78.44%) (6). This underscores the critical role of adolescents as key reservoirs of infection. Supporting this, the 2022 pertussis report from European union countries revealed that 70% of reported cases were individuals aged 14 years and older, while only 6% were infants under one year of age (7).

Critically, studies indicate that China’s pertussis burden is substantially underestimated, particularly among older children, adolescents, and adults, prompting calls for expanded booster vaccination in these groups (8). Susceptibility spans all ages, with unvaccinated infants, immunocompromised individuals, and infants under 6 months at highest risk due to ineffective maternal antibody protection (9, 10). These findings underscore the urgent need for enhanced surveillance, targeted immunization strategies, and public awareness to mitigate transmission and protect vulnerable populations.

Currently, three main types of combination vaccines for preventing pertussis, diphtheria, and tetanus are widely licensed and used in many countries: the diphtheria, tetanus, and acellular pertussis combined vaccine (DTaP); the diphtheria, tetanus, and acellular pertussis-haemophilus influenzae type b combined vaccine (DTaP-Hib); and the diphtheria, tetanus, and acellular pertussis-inactivated poliovirus-haemophilus influenzae type b combined vaccine (DTaP-IPV/Hib) (11). In the market, Boostrix and Adacel are most used, and the pertussis antigens in the former are PT, FHA, and PRN, in the later are PT, FHA, PRN, and FIM 2&3 (12, 13). However, Boostrix, Adacel and other reduced pertussis-containing vaccines have not yet been licensed for use in China. From 1978 to 2024, China’s immunization program established DTaP administration as 4 doses, with 3 months, 4 months, 5 months, and booster 18 months. Since January 1, 2025, the DTaP immunization schedule has been adjusted to 2 months, 4 months, 6 months, 18 months, and an additional dose DTaP (fifth dose) at 6 years of age (14). Therefore, there are only approved pertussis-containing vaccine for use in children aged 6 years and below, leaving a significant gap in immunization for older populations, and currently no licensed pertussis-containing vaccines specifically designed for adolescents and adults in China, highlighting an urgent need to address this unmet public health demand.

Here, we report on the development of the Tetanus, Reduced Diphtheria, and Acellular Pertussis (Five Components) Combined Vaccine, Adsorbed (Tdcp), by CanSino Biologics Inc. This vaccine is produced using *Corynebacterium diphtheriae*, *Clostridium tetani*, and genetically engineered *Bordetella pertussis* strains, including pertussis toxoid (PT), filamentous hemagglutinin (FHA), and pertactin (PRN) production strains. The Tdcp vaccine is a combination vaccine containing seven active ingredients: FHA, PT, PRN, fimbriae 2&3 (FIM2&3), diphtheria toxoid (DT), and tetanus toxoid (TT). It is designed for active immunization to protect against pertussis, diphtheria, and tetanus in individuals aged 6 years and older. This study aims to evaluate the safety and immunogenicity of the Tdcp vaccine produced by CanSino Biologics in participants aged 6 years and above. Notably, Tdcp vaccine is the first and only domestically developed product approved for clinical use in China.

## Method

### Study design

This is a randomised, blinded, controlled phase I trial, sited in Shangluo, Shaanxi. Eligible participants aged above 6 years old were recruited and divided into three age subgroups: participants in 12–17 years old and ≥18 years old groups (have not been vaccinated with any component of diphtheria, tetanus and pertussis vaccine within 5 years) were randomised to receive Tdcp or 23-valent pneumococcal polysaccharide vaccine (PPV23) in a 4:1 ratio; participants in 6–11 years old groups (only completed 4 doses of vaccines containing diphtheria, tetanus and pertussis components but have not received the fifth dose, with an interval of ≥ 3 years from the fourth dose) were randomised to receive Tdcp or Diphtheria and Tetanus Combined Vaccine, Adsorbed (DT) in a 1:1 ratio. In each group, 5 ml of blood and 5 ml of urine were collected before and on the 4th day after vaccination for blood routine and blood biochemistry examination, and urinalysis respectively. Besides, approximately 5 ml blood samples were collected before and on the 30th day after vaccination for immunogenicity analysis. Exclusion criteria included axillary temperature >37.0 °C; having suffered from diphtheria or tetanus, and pertussis in the last three years; volunteers aged ≥12 years who have been vaccinated with a vaccine containing the pneumococcal polysaccharide/conjugate component within 4 years; having had household contact with an individual diagnosed with pertussis, diphtheria or tetanus in the past 30 days; history of convulsions, epilepsy, encephalopathy and severe neurological disorders (e.g., transverse myelitis, Guillain-Barre syndrome, demyelinating disorders, etc.) and so on.

The primary endpoint was the incidence of adverse reactions 0–30 day after vaccination. The secondary safety endpoints were the incidence of serious adverse events (SAEs) 360 days after vaccination in the 6~11 years old group, and incidence of SAEs 180 days after vaccination in the 12–17 years old group and ≥18 years old group. Immunogenicity endpoints include antibody seroconversion rate and geometric mean concentration (GMC) of serum anti-DT, TT, PT, FHA, PRN, FIM 2&3–30 days after vaccination.

This study was registered on clinical trial, NCT06056050. The protocol was approved by the ethics committee of Shaanxi Provincial Centre for Disease Control and Prevention (2023-001-03, approved on 2023-08-18). The trial was carried out in accordance with the “Good Clinical Practice” (GCP) of the National Medical Products Administration (NMPA), and Declaration of Helsinki. Written informed consent has been obtained before screening.

### Luminx

The antibody responses to pertussis (PT, FHA, PRN and FIM 2&3), diphtheria, and tetanus antigens were quantitatively measured using a multiplexed fluorescent immunoassay based on Luminex technology. This high-throughput methodology employs antigen-conjugated magnetic microspheres, where each bead region is uniquely coded and coated with specific vaccine antigens. Reference sera were traceable to the 1st International Reference Preparation 06/140, and the national human antiserum reference material of China was used, with coated antigens sourced from the national reference antigens of China. During the assay, serum samples were incubated with the mixed antigen-coupled beads, allowing specific antibodies to bind to their respective antigens. Following washing steps to remove unbound components, phycoerythrin-conjugated detection antibodies were introduced to form fluorescent immune complexes. The Luminex analyser simultaneously identified each microsphere region and quantified the bound antibodies. Standard curves were generated using an 8-point dilution series of reference sera, with data analysis performed five-parameter logistic regression. Each analytical run included one high-level and one low-level quality control serum, and the coefficient of variation for quality control sera was required to be less than 20%. All samples were tested at three optimised dilutions in duplicate, with geometric mean antibody concentrations calculated after excluding outliers demonstrating >20% coefficient of variation between replicates. Samples with antibody concentrations outside the quantitative range were re-tested at appropriate dilutions to ensure accurate measurement.

### Vaccine

The Tdcp produced by CanSino Biologics. is prepared by mixing and diluting the original solution of pertussis FHA, PT, PRN, FIM 2&3, DT, and TT in the appropriate proportions with the addition of aluminium adjuvant. 0.5 ml was intramuscularly administrated in the lateral deltoid muscle of the upper arm. There were two control vaccines. One is PPV23 produced by Chengdu institute of biological products Co., Ltd., with 0.5ml per vial. Another one is Diphtheria and Tetanus Combined Vaccine, Adsorbed (DT) produced by Wuhan institute of biological products Co., Ltd., with 2.0 ml per ampoule.

The trial was a blinded study for participants and researchers. In this study, since the specifications of the different test drugs varied, participants, project statisticians and laboratory testing personnel were blinded, except for vaccination room personnel who were unblinded. Non-blinded personnel were not involved in field work other than that described above, and all other study personnel remained blinded.

### Statistical analysis

Safety analysis was based on the safety set (SS). The number of cases of adverse events and adverse reactions were counted and the incidence of adverse events and adverse reactions were calculated for each age group of the participant population, and the description of the grades of adverse events and adverse reactions was based on the constitutive ratios. The chi-square test or exact probability method was used to compare the differences in the incidence of adverse events and adverse reactions, grade 3 adverse events and adverse reactions, and SAEs between the experimental and control groups in each age group.

Confidence interval estimation was used to calculate the seroconversion rate and 95% CI, GMC and 95% CI of anti-PT, FHA, PRN, FIM 2&3, DT and TT antibodies in each group after immunization.

As for seroconversion rate criteria, for anti-DT and TT antibodies, pre-vaccination antibody concentration <0.1 IU/ml, post-vaccination antibody concentration ≥0.4 IU/ml; pre-vaccination antibody concentration ≥0.1 IU/ml and <2.0 IU/ml, 4-fold increase in post-vaccination antibody concentration; pre-vaccination antibody concentration ≥2.0 IU/ml, 2-fold increase in post-vaccination antibody concentration was regarded as seroconversion. For anti-PT, FHA, and PRN antibodies, if the concentration of pre-vaccination antibody was <5 IU/ml, the concentration of post-vaccination antibody was ≥20 IU/ml; if the concentration of pre-vaccination antibody was >5 IU/ml but <20 IU/ml, the concentration of post-vaccination antibody increased 4-fold; if the concentration of pre-vaccination antibody was ≥20 IU/ml, the concentration of post-vaccination antibody increases 2-fold, and it was considered to be a seroconversion. For anti-FIM2&3 antibodies, if the concentration of pre-vaccination antibody was <5 EU/ml, the concentration of post-vaccination antibody was ≥20 EU/ml; if the concentration of pre-vaccination antibody was >5 EU/ml but <20 EU/ml, the concentration of post-vaccination antibody increased 4-fold; if the concentration of pre-vaccination antibody was ≥20 EU/ml, the concentration of post-vaccination antibody increased 2-fold, which was considered to be a seroconversion.

## Results

**Figure 1:**
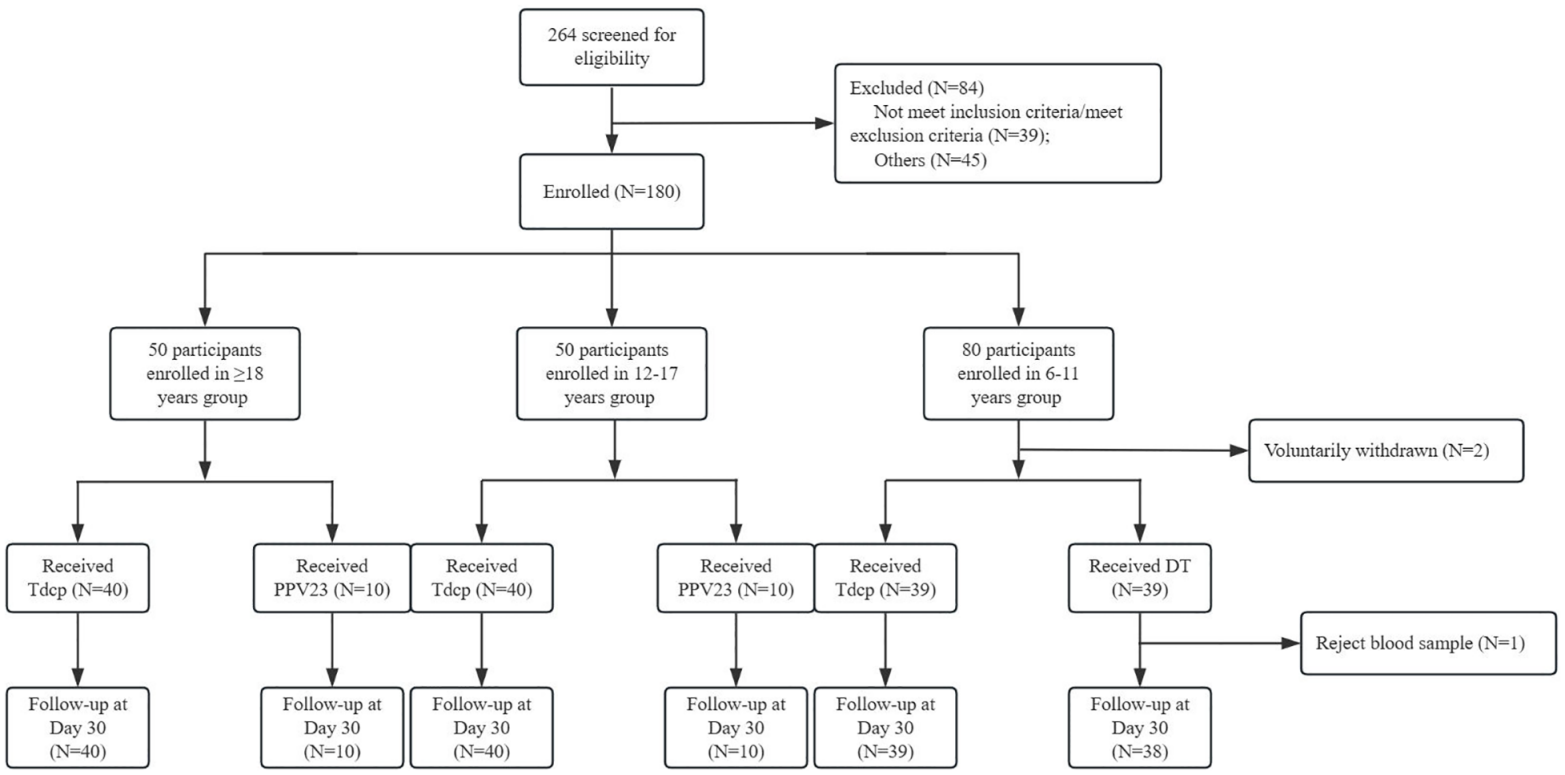
CONSORT flow diagram of the randomised trial.

The demographic characteristics of each age group were shown in **Table 1**. There were 10 males and 30 females in the ≥18 years experimental group, with a mean age of 42.35 years, and 4 males and 6 females in the control group, with a mean age of 42.90 years. 26 males and 14 females in the 12–17 years experimental group, with a mean age of 13.40 years, and 9 males and 1 female in the control group, with a mean age of 13.10 years. 24 males and 15 females in the 6–11 years experimental group, with a mean age of 6.10 years, and 17 males and 22 females in the control group, with a mean age of 6.10 years. The age and gender composition of the experimental and control groups was balanced in each age group, and the differences between the age, gender, and BMI groups were not statistically significant (*p >*0.05). In this study, 264 volunteers were screened, 180 participants were finally enrolled, and 178 participants completed one dose of vaccination. A total of 2 subjects voluntarily withdrawn (**Figure 1**).

**Table 1:** Demographic and baseline characteristics (SS).

	≥18 years group			12–17 years group			6–11 years group		
	Tdcp (N = 40)	PPV23 (N = 10)	*p*	Tdcp (N = 40)	PPV23 (N = 10)	*p*	Tdcp (N = 39)	DT (N = 39)	*p*
Age, years	42.35 (10.62)	42.90 (7.19)	0.627	13.40 (1.13)	13.10 (0.74)	0.488	6.10 (0.64)	6.10 (0.50)	0.589
Gender, female (n)	30 (75.00)	6 (60.00)	0.581	14 (35.00)	1 (10.00)	0.247	15 (38.46)	22 (56.41)	0.112
Male	10 (25.00)	4 (40.00)		26 (65.00)	9 (90.00)		24 (61.54)	17 (43.59)	
BMI, kg/m^2^	24.97 (3.46)	25.93 (2.08)	0.405	20.03 (3.39)	21.08 (3.29)	0.259	16.46 (1.77)	16.65 (2.51)	0.920

### Safety

**Table 2** presented the incidence of adverse reactions occurring within 0–30 days after vaccination, stratified by age groups and vaccine types. For each age group, the adverse reactions are compared between different vaccines, and the *p*-values are provided to indicate the statistical significance of the differences observed. In the ≥18 years group, 70.00% of Tdcp recipients reported adverse reactions compared to 40.00% of PPV23 recipients (*p* = 0.162). In the 12–17 years group, 50.00% of Tdcp recipients reported adverse reactions compared to 30.00% of PPV23 recipients (*p* = 0.435). In the 6–11 years group, 43.59% of both Tdcp and DT recipients reported adverse reactions (*p*>0.999). Pain was more commonly reported in the ≥18 years group for Tdcp (55.00%) compared to PPV23 (30.00%) (*p* = 0.157), while in the 12–17 years group, pain was equally reported (30.00%) for both vaccines (*p*>0.999). In the 6–11 years group, pain was reported by 20.51% of Tdcp recipients and 12.82% of DT recipients (*p* = 0.362). Other adverse reactions, such as itching, induration, swelling, and erythema, were reported at varying rates across the groups, with no statistically significant differences in most cases. Systemic reactions like decreased appetite, cough, fever, fatigue, nausea, and diarrhoea were less common and generally did not show significant differences between vaccine groups.

**Table 2:** Incidence of adverse reactions 0–30 day after vaccination.

	≥18 years group			12–17 years group			6–11 years group		
	Tdcp (N = 40)	PPV23 (N = 10)	*p*	Tdcp (N = 40)	PPV23 (N = 10)	*p*	Tdcp (N = 39)	DT (N = 39)	*p*
Total adverse reactions	28 (70.00%)	4 (40.00%)	0.162	20 (50.00)	3 (30.00%)	0.435	17 (43.59%)	17 (43.59)	>0.999
Pain	22 (55.00%)	3 (30.00%)	0.157	12 (30.00%)	3 (30.00%)	>0.999	8 (20.51%)	5 (12.82%)	0.362
Itching	13 (32.50%)	1 (10.00%)	0.306	8 (20.00%)	0	0.289	8 (20.51%)	9 (23.08)	0.784
Induration	7 (17.50%)	1 (10.00%)	0.923	4 (10.00%)	0	0.571	4 (10.26%)	3 (7.69%)	>0.999
Swelling	6 (15.00%)	0	0.446	3 (7.50%)	0	>0.999	3 (7.69%)	4 (10.26%)	>0.999
Erythema	2 (5.00%)	0	>0.999	1 (2.50%)	0	>0.999	1 (2.56%)	3 (7.69%)	0.608
Decreased appetite	1 (2.50%)	0	>0.999	1 (2.50%)	0	>0.999			–
Cough	1 (2.50%)	0	>0.999			–	0	1 (2.56%)	>0.999
Fever	1 (2.50%)	1 (10.00%)	0.363	1 (2.50%)	0	>0.999	3 (7.69%)	3 (7.69%)	>0.999
Fatigue	1 (2.50%)	0	>0.999			–	1 (2.56%)	2 (5.13%)	>0.999
Nausea	5 (12.50)	0	0.556			–			–
Diarrhoea			–	0	1 (10.00%)	0.200			–

Within 0–30 days after vaccination, the incidence of adverse reaction in above 18 years group showed that decreased appetite, cough, fatigue, and nausea occurred only in the experimental group, with incidence rates of 2.50%, 2.50%, 2.50%, and 12.50%, respectively. The incidence of fever was 2.50% and 10.00% in the experimental and control groups, respectively. For local adverse reaction, erythema and swelling occurred only in the experimental group with an incidence of 5.00% and 15.00% respectively. The incidence of induration was 17.50% and 10.00% respectively, and the incidence of itching was 32.50% and 10.00% respectively.

As for 12–17 years group, local adverse reaction erythema, induration, swelling, itching occurred only in the experimental group, with an incidence of 2.50%, 10.00%, 7.50%, and 20.00%, respectively, and the incidence of in the experimental group and the control group was 30.00% and 30.00%, respectively. Systemic adverse reactions were decreased appetite, fever, and diarrhoea, of which decreased and fever occurred only in the experimental group with an incidence rate of 2.50%, and diarrhoea occurred only in the control group with an incidence rate of 10.00%.

Within 0–30 days after vaccination, the local adverse reactions in the 6–11 years group were erythema, pain, induration, swelling and itching, of which the incidence of erythema in the experimental group and the control group was 2.56% and 7.69%, the incidence of induration was 10.26% and 7.69%, the incidence of swelling was 7.69% and 10.26%, and the incidence of itching was 20.51% and 23.08%. The systemic adverse reactions were cough, fever, and fatigue, of which cough occurred only in the control group with an incidence rate of 2.56%, fever incidence in the experimental group and the control group was 7.69% and 7.69%, and fatigue incidence was 2.56% and 5.13%, respectively.

Within 0–30 days after vaccination, adverse reactions occurred within 7 days in both the ≥18 years experimental group and the control group, with an incidence of 70.00% and 40.00%, respectively. The difference between the groups was not statistically significant (*p* = 0.162). No adverse reactions occurred within 8–30 days. Adverse reactions in the 12–17 years experimental group and control group were also within 7 days, and the incidence rate was 50.00% and 30.00%, respectively. The difference between the groups was not statistically significant (*p* = 0.435). No adverse reactions occurred within 8–30 days. Within 0~30 days after vaccination, the time of occurrence of adverse reactions in both the 6–11 years experimental group and the control group was within 7 days, with an incidence rate of 43.59% and 43.59%, respectively. There is no significant difference between the groups (*p*>0.999). No adverse reactions occurred within 8–30 days (**Table 3**).

**Table 3:** Duration of adverse reactions 0–30 days after vaccination.

	≥18 years group			12–17 years group			6–11 years group		
	Tdcp (N = 40)	PPV23 (N = 10)	*p*	Tdcp (N = 40)	PPV23 (N = 10)	*p*	Tdcp (N = 39)	DT (N = 39)	*p*
Total									
0-7d	70.00%	40.00%	0.162	50.00%	30.00%	0.435	43.59%	43.59%	>0.999
0-30d	70.00%	40.00%	0.162	50.00%	30.00%	0.435	43.59%	43.59%	>0.999
Solicited									
0-7d	70.00%	40.00%	0.162	47.50%	30.00%	0.522	43.59%	41.03%	0.819
0-30d	70.00%	40.00%	0.162	47.50%	30.00%	0.522	43.59%	41.03%	0.819
Local									
0-7d	67.50%	40.00%	0.216	42.50%	30.00%	0.718	41.03%	33.33%	0.482
0-30d	67.50%	40.00%	0.216	42.50%	30.00%	0.718	41.03%	33.33%	0.482
Systemic									
0-7d	27.50%	10.00%	0.456	5.00%	10.00%	0.496	7.69%	15.38%	0.478
0-30d	27.50%	10.00%	0.456	5.00%	10.00%	0.496	7.69%	15.38%	0.478

### Immunogenicity

**Table 4** illustrated seroconversion rates of anti-DT, TT, PT, FHA, PRN, and FIM 2&3 antibodies at 30 days post-vaccination demonstrated significant differences between the Tdcp experimental groups and control groups (PPV23 or DT) across three age cohorts (≥18 years, 12–17 years, and 6–11 years). In the ≥18 years cohort, the Tdcp group demonstrated significantly higher seroconversion rates compared to the PPV23 control group. Anti-PT and anti-PRN antibodies achieved 100.00% seroconversion rate, with anti-FHA, anti-FIM 2&3 and anti-TT antibodies reaching 97.50%, 95.00% and 77.50%, respectively (all *p* < 0.001). Anti-DT antibody seroconversion was moderate at 60.00% but remained significantly higher than the control group (*p* = 0.002). In the 12–17 years cohort, the Tdcp group exhibited robust immunogenicity, with anti-TT, anti-PT, anti-FHA, and anti-FIM 2&3 antibodies achieving 100.00% seroconversion rate (all *p* < 0.001). Anti-PRN and anti-DT antibodies also showed high seroconversion rates of 97.50% and 82.50%, respectively (both *p* < 0.001). These results highlight the strong immune response elicited by the Tdcp vaccine in adolescents. For the 6–11 years cohort, significant differences were observed between the Tdcp and DT control groups. Anti-PT, anti-FHA, anti-PRN, and anti-FIM 2&3 antibodies demonstrated substantially higher seroconversion rates in the Tdcp group (79.49–100.00%) compared to the control group (0–5.26%, all *p* < 0.001). Anti-TT seroconversion was 100.00% in the Tdcp group versus 78.95% in the control group (*p* = 0.008). However, no significant difference was observed for anti-DT antibodies (94.87% vs. 100.00%, *p* = 0.494), likely due to the high baseline immunogenicity of the DT vaccine in this age group. Overall, the Tdcp vaccine induced significantly higher seroconversion rates across all target antibodies compared to the control groups, particularly for pertussis-related antigens (PT, FHA, PRN, and FIM 2&3). The lack of difference in anti-DT responses among younger children underscores the inherent efficacy of standalone DT immunization in this population.

**Table 4:** Anti-DT, TT, PT, FHA, PRN, FIM 2&3 antibody seroconversion rate 30days after vaccination.

Antibody	≥18 years group			12–17 years group			6–11 years group		
	Tdcp (N = 40)	PPV23 (N = 10)	*p*	Tdcp (N = 40)	PPV23 (N = 10)	*p*	Tdcp (N = 39)	DT (N = 38)	*p*
Anti-PT	100.00 (91.19,100.00)	0 (0.00,30.85)	<0.001	100.00 (91.19,100.00)	0 (0.00,30.85)	<0.001	79.49 (63.54,90.70)	2.63 (0.07,13.81)	<0.001
Anti-FHA	97.50 (86.84,99.94)	0 (0.00,30.85)	<0.001	100.00 (91.19,100.00)	0 (0.00,30.85)	<0.001	92.31 (79.13,98.38)	2.63 (0.07,13.81)	<0.001
Anti-PRN	100.00 (91.19,100.00)	0 (0.00,30.85)	<0.001	97.50 (86.84,99.94)	0 (0.00,30.85)	<0.001	100.00 (90.97,100.00)	0 (0.00,9.25)	<0.001
Anti-FIM 2&3	95.00 (83.08,99.39)	0 (0.00,30.85)	<0.001	100.00 (91.19,100.00)	0 (0.00,30.85)	<0.001	87.18 (72.57,95.70)	5.26 (0.64,17.75)	<0.001
Anti-DT	60.00 (43.33,75.14)	0 (0.00,30.85)	0.002	82.50 (67.22,92.66)	0 (0.00,30.85)	<0.001	94.87 (82.68,99.37)	100.00 (90.75,100.00)	0.494
Anti-TT	77.50 (61.55,89.16)	0 (0.00,30.85)	<0.001	100.00 (91.19,100.00)	0 (0.00,30.85)	<0.001	100.00 (90.97,100.00)	78.95 (62.68,90.45)	0.008

**Table 5** showed the geometric mean concentrations (GMCs) of anti-DT, TT, PT, FHA, PRN, and FIM 2&3 antibodies were evaluated pre- and 30 days post-vaccination in the Tdcp experimental groups and control groups (PPV23 or DT) across three age cohorts (≥18 years, 12–17 years, and 6–11 years). In the ≥18 years cohort, pre-vaccination GMCs for all antibodies were low, with no significant differences between the Tdcp and PPV23 groups except for anti-DT antibodies (0.02 vs. 0.01, *p* = 0.041). At 30 days post-vaccination, the Tdcp group showed significantly higher GMCs for all antibodies compared to the control group: anti-DT (0.52 vs. 0.03, *p* < 0.001), anti-TT (1.51 vs. 0.05, *p* < 0.001), anti-PT (125.60 vs. 5.20, *p* < 0.001), anti-FHA (190.53 vs. 17.71, *p* < 0.001), anti-PRN (551.03 vs. 7.78, *p* < 0.001), and anti-FIM 2&3 (415.05 vs. 3.22, *p* < 0.001). In the 12–17 years cohort, pre-vaccination GMCs were similarly low, with no significant differences between the Tdcp and PPV23 groups for any antibody. Post-vaccination, the Tdcp group exhibited significantly higher GMCs for all antibodies: anti-DT (1.76 vs. 0.07, *p* < 0.001), anti-TT (11.15 vs. 0.07, *p* < 0.001), anti-PT (150.28 vs. 6.27, *p* < 0.001), anti-FHA (162.82 vs. 20.14, *p* = 0.002), anti-PRN (673.38 vs. 16.63, *p* < 0.001), and anti-FIM 2&3 (702.89 vs. 8.34, *p* < 0.001). For the 6–11 years cohort, pre-vaccination GMCs were comparable between the Tdcp and DT groups, with no significant differences. At 30 days post-vaccination, the Tdcp group demonstrated significantly higher GMCs for anti-TT (7.59 vs. 1.69, *p* < 0.001), anti-PT (131.14 vs. 10.84, *p* < 0.001), anti-FHA (176.84 vs. 8.66, *p* < 0.001), anti-PRN (1074.07 vs. 37.99, *p* < 0.001), and anti-FIM 2&3 (555.49 vs. 18.46, *p* < 0.001). However, no significant difference was observed for anti-DT antibodies (4.33 vs. 4.92, *p* > 0.05), likely due to the robust immunogenicity of the DT vaccine in this age group. The Tdcp vaccine induced significantly higher post-vaccination GMCs for all target antibodies compared to the control groups, except for anti-DT in the 6–11 years cohort. These results underscore the strong immunogenicity of the Tdcp vaccine, particularly for pertussis-related antigens (PT, FHA, PRN, and FIM 2&3), across all age groups.

**Table 5:** Anti-DT, TT, PT, FHA, PRN, FIM 2&3 antibody GMC.

		≥18 years group			12–17 years group			6–11 years group		
Antibody		Tdcp (N = 40)	PPV23 (N = 10)	*p*	Tdcp (N = 40)	PPV23 (N = 10)	*p*	Tdcp (N = 39)	DT (N = 38)	*p*
Anti-PT	Pre-vaccination	4.80 (2.89)	4.34 (4.76)	0.807	5.96 (2.77)	6.62 (9.52)	0.888	11.56 (6.79)	9.34 (4.07)	0.580
	30 days post-vaccination	125.60 (2.00)	5.20 (3.67)	<0.001	150.28 (2.11)	6.27 (6.49)	<0.001	131.14 (2.12)	10.84 (3.81)	<0.001
Anti-FHA	Pre-vaccination	15.11 (3.11)	15.81 (2.50)	0.908	15.68 (2.90)	23.06 (4.68)	0.356	9.20 (7.07)	7.63 (7.03)	0.676
	30 days post-vaccination	190.53 (1.81)	17.71 (2.40)	<0.001	162.82 (1.97)	20.14 (4.62)	0.002	176.84 (3.00)	8.66 (6.64)	<0.001
Anti-PRN	Pre-vaccination	6.23 (4.29)	3.36 (4.37)	0.237	16.40 (4.27)	14.93 (5.45)	0.861	40.20 (2.91)	35.57 (2.57)	0.595
	30 days post-vaccination	551.03 (3.25)	7.78 (2.48)	<0.001	673.38 (2.46)	16.63 (4.61)	<0.001	1074.07 (1.87)	37.99 (2.68)	<0.001
Anti-FIM 2&3	Pre-vaccination	4.27 (5.21)	2.09 (4.56)	0.221	13.94 (4.44)	9.07 (7.18)	0.449	21.43 (10.64)	17.23 (6.41)	0.654
	30 days post-vaccination	415.05 (4.61)	3.22 (3.73)	<0.001	702.89 (2.64)	8.34 (5.55)	<0.001	555.49 (2.39)	18.46 (7.21)	<0.001
Anti-DT	Pre-vaccination	0.02 (3.49)	0.01 (3.99)	0.041	0.03 (4.14)	0.07 (3.56)	0.101	0.14 (2.71)	0.11 (3.25)	0.111
	30 days post-vaccination	0.52 (7.52)	0.03 (2.16)	<0.001	1.76 (3.91)	0.07 (3.27)	<0.001	4.33 (2.19)	4.92 (2.50)	0.373
Anti-TT	Pre-vaccination	0.02 (2.85)	0.01 (2.73)	0.742	0.05 (3.83)	0.08 (2.74)	0.392	0.15 (4.39)	0.17 (3.11)	0.784
	30 days post-vaccination	1.51 (5.61)	0.05 (1.69)	<0.001	11.15 (2.29)	0.07 (2.50)	<0.001	7.59 (2.28)	1.69 (1.87)	<0.001

## Discussion

Immunity to pertussis does not last a lifetime, regardless of whether it is acquired through infection or vaccination. Adolescents and adults with waning immunity are prone to contracting pertussis, and family members can serve as a source for transmitting the disease to unvaccinated or incompletely vaccinated infants, who are at risk of developing severe complications from pertussis. In China, the number of reported pertussis cases decreased significantly from 30,027 cases in 2019 to 4,475 cases in 2020. However, with the COVID-19 pandemic under control, 9,961 cases of pertussis were reported in China in 2021, making it evident that the recurrence of pertussis is still a long-term tendency in China (15). Other studies indicated, 76% to 83% of whooping cough cases in infants and young children come from transmission within the family, suggesting widespread underdiagnosed infections and potential transmission chains within households (16). In the United States, it is estimated that approximately 600,000 cases of pertussis occur in adults each year. The necessity of preventing pertussis in older age groups and reducing the likelihood of transmission from older individuals to susceptible infants has guided the formulation of policies for the routine immunization of adolescents and adults with reduced antigen content acellular pertussis-containing vaccines (17).

The incidence of total adverse reactions in the experimental group of subjects aged 6 years and older was 54.62%, with local adverse reactions being the predominant type. Common local adverse reactions included erythema, pain, induration, swelling, and itching, which occurred across all age groups. Among these, pain and itching were the most frequently reported, with incidence rates exceeding 20% in all age experimental groups. Notably, in adults (≥18 years), the incidence of pain was comparable to that observed in Boostrix and Adacel studies (17). Specifically, injection site pain was reported in 61.0% of Boostrix recipients and 69.2% of Adacel recipients, while the experimental group showed a similar trend, further confirming the localised nature of these reactions. In adolescents (12–17 years), pain incidence was also consistent with Boostrix and Adacel data, highlighting the predictable and manageable profile of local reactions. Systemic adverse reactions were generally mild, with fever being one of the most common symptoms across all experimental groups. In the ≥18 years group, nausea occurred at an incidence of 12.50%, while other systemic symptoms such as fatigue and headache were reported at rates below 10% in all age groups. Importantly, total adverse reactions in subjects aged 6 years and above were predominantly grade 1 and 2, with grade 3 reactions being rare and limited to fever. When comparing the 6–11 years Tdcp group to DT group, the addition of the acellular pertussis component did not significantly increase the incidence of adverse reactions. In fact, the safety profile of the experimental vaccine was comparable to that of DT, with no additional safety concerns attributed to the pertussis component. This suggested that the inclusion of acellular pertussis antigens maintains a well-tolerated safety profile while providing broader protection against pertussis.

The analysis of anti-DT, TT, PT, FHA, PRN, and FIM 2&3 antibody results showed that on the 30th day after vaccination, the GMC of each antibody in the experimental groups increased significantly. There was no significant change in the levels of each antibody on the 30th day post-vaccination in the ≥18 years and 12–17 years control groups. In the 6–11 years control group, the GMC and seroconversion rate of anti-DT and TT antibodies increased significantly on the 30th day post-vaccination, while the rest of the antibody levels did not change significantly after vaccination. Notably, the GMC of anti-PT antibodies in the experimental groups (Tdcp) was significantly higher than that observed in both the Boostrix and Adacel vaccine groups, which typically reported anti-PT GMCs lower than 100 IU/mL. This suggested a stronger immune response to PT in the Tdcp group compared to these widely used vaccines (17).

The experimental vaccine had an anti-DT seroconversion rate of 60% in the ≥18 years group and all other antibody seroconversion rates were above 77.50%. In a study used Boostrix as experimental vaccine and DT as control vaccine, one month after the booster dose, 100% of dTpa recipients had seroprotective antibody concentrations against diphtheria and tetanus. In addition, increases in anti-pertussis antibody GMCs of between five and 18-fold were observed in the dTpa group after the booster dose, whereas no response was seen in recipients of the DT vaccine that does not contain pertussis antigens (18). However, it is important to note that China currently lacks a routine immunization program for DT vaccination in individuals aged 12 years and above. For adults, particularly those were primary immunization, resulting in lower seroconversion rates compared to populations with regular booster schedules. This gap in immunization coverage highlights the urgent need for DT booster vaccination among Chinese adults, as waning immunity increases the risk of diphtheria and tetanus infections in this demographic (19, 20). Moreover, the inclusion of Tdcp vaccines not only enhances protection against pertussis but also provides a valuable opportunity to strengthen diphtheria and tetanus immunity in adults. Given the rising incidence of pertussis in adolescents and adults, and their role as potential transmission sources to vulnerable infants, implementing Tdcp booster programs for adults could significantly improve overall population immunity and reduce disease burden.

The study was limited by small number of participants. This is a phase I study to assess the preliminary safety and immunogenicity of Tdcp, and a phase II/III trial will conduct to support the further application of Tdcp in large number of participants. Besides, since there was no pertussis-containing vaccine applied for children above 6 years in China, the PPV23 control groups were not positive group. From Jan 2025, the immunization schedule for DTaP vaccine in China has been adjusted to one dose at 2 months, 4 months, 6 months, 18 months, and 6 years of age, therefore, the positive control vaccine was used in 6 years group in further phase II/III trial (14). Overall, the results showed Tdcp was generally well tolerated, and no safety concerns were raised in this study. The GMC and seroconversion rate of each antibody in the experimental group could reach a high level, indicating that the experimental vaccine had good immunogenicity.

## Data Availability

The raw data supporting the conclusions of this article will be made available by the authors, without undue reservation.
